# Multiple Ligand-Bound States of a Phosphohexomutase Revealed by Principal Component Analysis of NMR Peak Shifts

**DOI:** 10.1038/s41598-017-05557-w

**Published:** 2017-07-13

**Authors:** Jia Xu, Akella V. S. Sarma, Yirui Wei, Lesa J. Beamer, Steven R. Van Doren

**Affiliations:** 1 Department of Biochemistry, 117 Schweitzer Hall, University of Missouri, Columbia, Missouri 65211 USA; 2 Centre for NMR, Indian Institute of Chemical Technology, Uppal Road, Hyderabad, 500 607 India

## Abstract

Enzymes sample multiple conformations during their catalytic cycles. Chemical shifts from Nuclear Magnetic Resonance (NMR) are hypersensitive to conformational changes and ensembles in solution. Phosphomannomutase/phosphoglucomutase (PMM/PGM) is a ubiquitous four-domain enzyme that catalyzes phosphoryl transfer across phosphohexose substrates. We compared states the enzyme visits during its catalytic cycle. Collective responses of *Pseudomonas* PMM/PGM to phosphosugar substrates and inhibitor were assessed using NMR-detected titrations. Affinities were estimated from binding isotherms obtained by principal component analysis (PCA). Relationships among phosphosugar-enzyme associations emerge from PCA comparisons of the titrations. COordiNated Chemical Shifts bEhavior (CONCISE) analysis provides novel discrimination of three ligand-bound states of PMM/PGM harboring a mutation that suppresses activity. Enzyme phosphorylation and phosphosugar binding appear to drive the open dephosphorylated enzyme to the free phosphorylated state, and on toward ligand-closed states. Domain 4 appears central to collective responses to substrate and inhibitor binding. Hydrogen exchange reveals that binding of a substrate analogue enhances folding stability of the domains to a uniform level, establishing a globally unified structure. CONCISE and PCA of NMR spectra have discovered novel states of a well-studied enzyme and appear ready to discriminate other enzyme and ligand binding states.

## Introduction

Conformational change and substrate binding play central roles in enzyme catalysis, where coexistence of multiple conformations is ubiquitous^1^. However, many studies have failed to observe conformational change upon ligand binding, presumably due to technical limitations such as crystal packing effects. NMR chemical shifts are useful to study protein-ligand interactions, can be measured accurately, and are sensitive to subtle changes of structure or dynamics^2^. Chemical shifts in solution can provide detailed information on interchanging enzyme conformations from the catalytic cycle under native-like conditions, and can be used for atomic-resolution study of equilibria relevant to catalysis.

**Figure 1:**
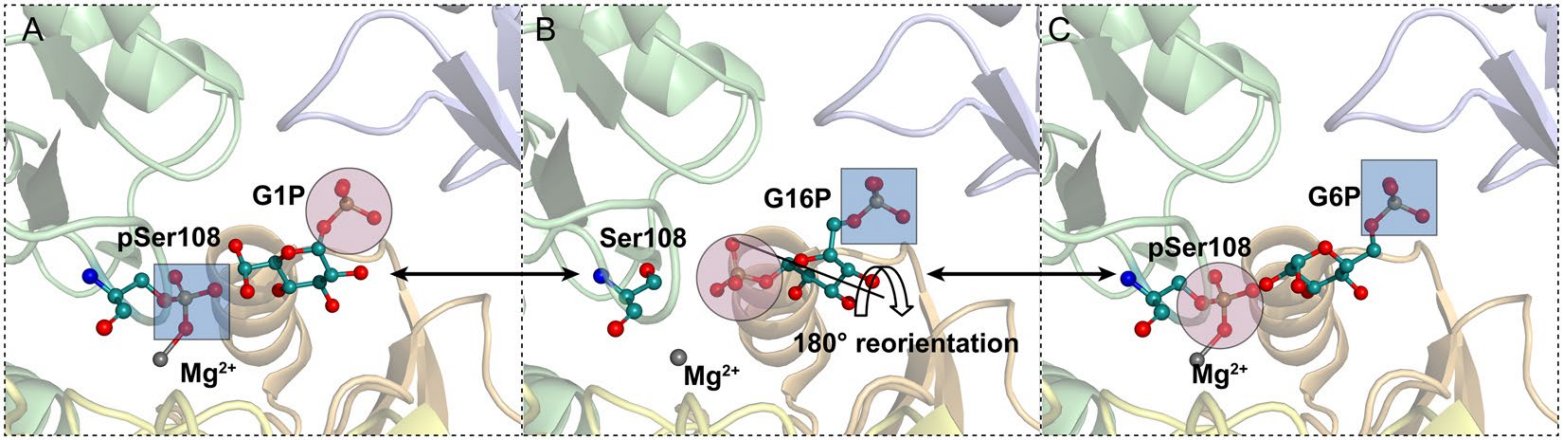
Reactions of PMM/PGM. The enzyme reversibly transforms G1P (**A**, PDB ID:1P5D) to G6P (**C**, PDB ID:1P5G) via an intermediate G16P, which undergoes 180° reorientation (**B**, PDB ID:2FKM). Domains 1 to 4 are colored pastel green, yellow, orange, or blue, in turn. This color code applies to other figures showing crystal structures.

Enzymes in the α-*D*-phosphohexomutase superfamily are ubiquitous in carbohydrate biosynthesis. The representative phosphomannomutase/phosphoglucomutase (PMM/PGM; 52 kDa) contributes to the virulence of infections by *Pseudomonas aeruginosa* in cystic fibrosis, chronic obstructive pulmonary disease, and burn injuries^3^. Bacterial PMM/PGM participates in biosynthesis of virulence factors such as lipopolysaccharide, rhamnolipids, Pel and Psl polysaccharides, and alginate^4, 5^. *Pseudomonas* PMM/PGM comprises 463 residues and four domains. It catalyzes a reversible phosphoryl group transfer across phosphoglucose or phosphomannose substrates, depending on the pathway. The enzyme requires Mg^2+^ and conserved phosphoserine 108 (pSer108) for activity^6^. Its reaction mechanism entails two successive phosphoryl transfer steps (Fig. 1). First, the phosphoryl group of pSer108 is transferred to the monophosphorylated substrate, forming a bisphosphorylated intermediate which reorients by 180° within the active site^7, 8^. Then the phosphoryl group is transferred back to Ser108 from the intermediate, regenerating the phospho-enzyme.

Phosphorylation of Ser108 in the catalytic cleft widely stabilizes PMM/PGM, while dephosphorylation tends to increase structural flexibility^9, 10^. Structural studies of ligand binding revealed that domain 4 rotates to close the catalytic cleft upon binding of phosphosugar substrates^6, 11^. Among the ligands, xylose 1-phosphate (X1P), a substrate analog inhibitor, shifts the conformational ensemble of PMM/PGM towards a closed and less flexible ligand-bound state^9^. Crystallographic structures show three conformations of the catalytic cleft: open without ligand, ligand-closed, and half-open with glucose 1,6-bisphosphate (G16P) bound to phospho-enzyme (E_P_), which is off-pathway due to the phosphorylation^11^. Crystallography failed to detect the global changes in flexibility of the protein due to phosphorylation of Ser108 that were revealed by hydrogen^9, 10^.

In protein titrations with ligands, NMR can be used to measure progressive shifting of peaks in the fast exchange regime^12^, which reflect population-weighted averages of the spectral contributions of the states in rapid equilibrium^2^. Principal component analysis (PCA) applied to ^1^H and ^15^N chemical shift changes can gain insight from the shifts in population due to pH, [ligand], or time^13–16^. PCA is a widely used tool of unsupervised statistics that can extract patterns and perform cluster analysis on large, complex data sets^17^. Allosteric networks that regulate activity and respond to binding were elegantly elucidated by PCA of NMR chemical shift perturbations by ligands or mutations, using a method called chemical shift covariance analysis (CHESCA)^18–20^. Another statistical approach to NMR chemical shifts called COordinated Chemical Shifts bEhavior (CONCISE) evaluates the collective (global) response of a protein to perturbations such as ligand binding, mutation, or post-translational modification^21^. The method filters out minor changes not associated with the principal conformational equilibrium shift. Thus, the ability of PCA to extract main patterns and remove noise supports powerful analyses of chemical shifts of proteins.

We sought to understand the responses of PMM/PGM to phosphosugar substrates, inhibitor, and phoshorylation status using CONCISE and PCA. We estimated affinities for the ligands with the aid of PCA of the NMR-detected titrations. We conducted a parallel study using PMM/PGM(S108C) because this mutation eliminates phosphorylation of the enzyme and impedes transformation of glucose 1-phosphate (G1P) to glucose 6-phosphate (G6P) and preserves the G1P for days^22^. The influence of the X1P inhibitor on enzyme stability relative to the free states was obtained for individual residues and domains using NMR-detected hydrogen exchange. These studies revealed a progression of enzyme changes from the most open and least stable free state to complexes with substrates or inhibitor, distinguished by progressively higher affinities, CONCISE scores, and clustering by PCA. The structure of the enzyme responds globally to the perturbations, with the greatest responses found at the interface between domain 3 (D3) and domain 4 (D4) which is more mobile and closes the catalytic cleft.

## Results

### Ligands affect binding site and a domain-domain interface

**Figure 2:**
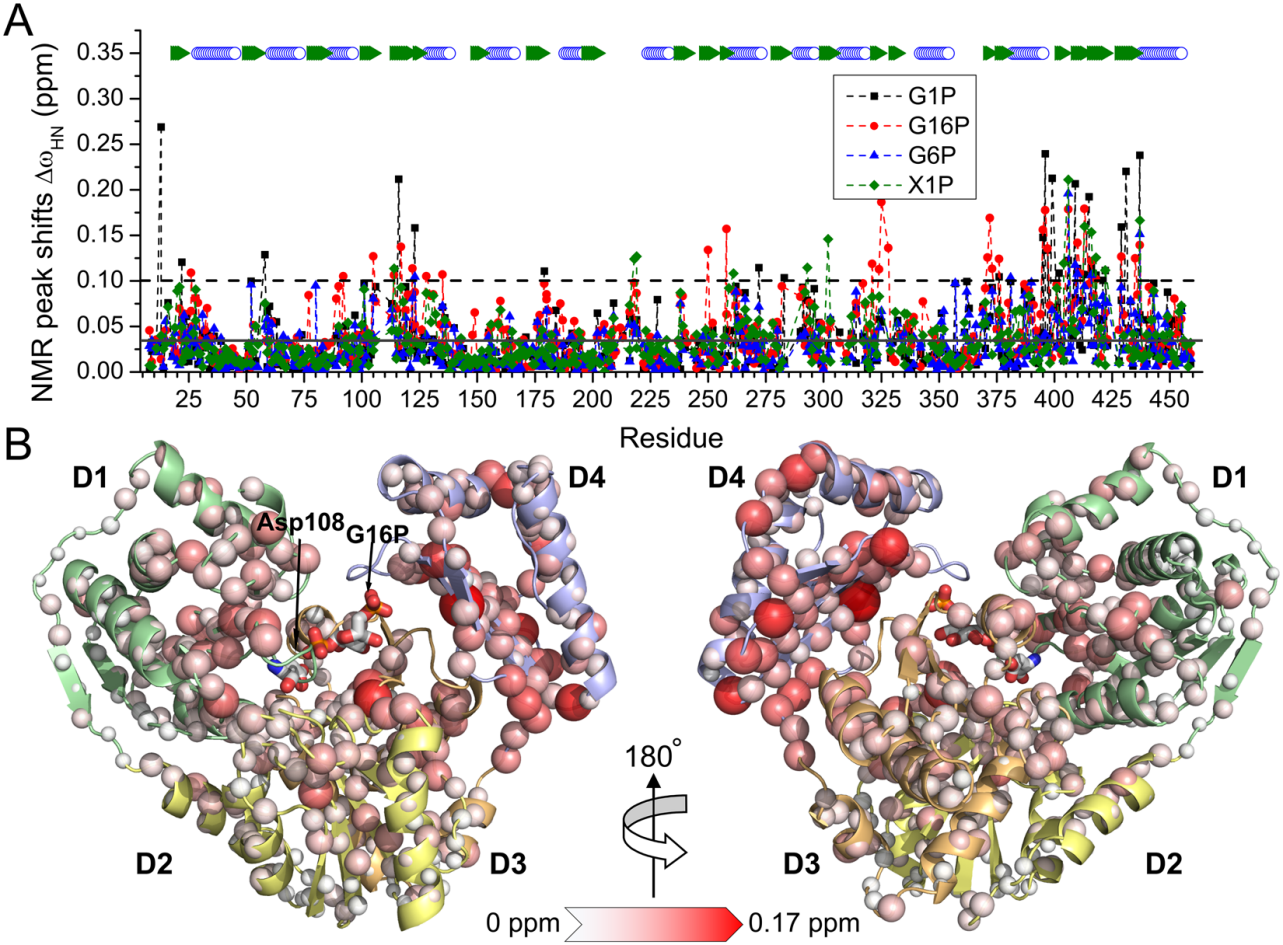
Residues of PMM/PGM(S108C) with NMR peaks shifted by ligand binding. (**A**) Amide ^1^H/^15^N NMR peak shifts are plotted using Eq. 1 for saturating additions of G1P (black squares), G16P (red circles), G6P (blue triangles), and X1P (green diamonds). The mean NMR peaks shifts are marked by a black line, while the dashed line represents threshold that is two σ above the average. (**B**) Mean NMR peak shift changes introduced by multiple ligand bindings are marked with spheres on the crystal structure of the G16P complex with PMM/PGM(S108D) (PDB ID:2FKM). The magnitudes of NMR peak shifts are represented by the white to red color gradient and by the radii of the spheres.

Phosphosugars were titrated into PMM/PGM from *P. aeruginosa*, either wild-type or with S108C mutation to impede catalytic turnover^22^. ^15^N TROSY NMR spectra of the mixtures were acquired at 800 MHz (Figs S1–S3). Assignments of the amide chemical shifts^22^ were updated for the complexes using the incremental peak shifts during the titrations (mostly undergoing the averaging of fast exchange); see Table S1 for the extent of the assignments. The phosphosugars shifted the backbone amide NMR peaks in similar locations in the enzyme (Fig. 2A and Supplementary Fig. S4). The largest chemical shift perturbations (CSPs) are observed around residues 15–28 and 100–135 in domain 1 (D1), 218–239 in domain 2 (D2), 258–328 in D3, and most of D4. Mapping of the averages of these peaks shifts onto D1 to D3 of the crystal structure shows that the residues with the most significantly shifted NMR peaks are located in the vicinity of the sugar-phosphate binding site (Fig. 2B). The large CSPs in D4 agree with the finding that this domain rotates upon ligand binding^11^. The most affected positions reside at the face of D4 nearest to the active site and D3.

### X1P exhibits highest affinity

**Figure 3:**
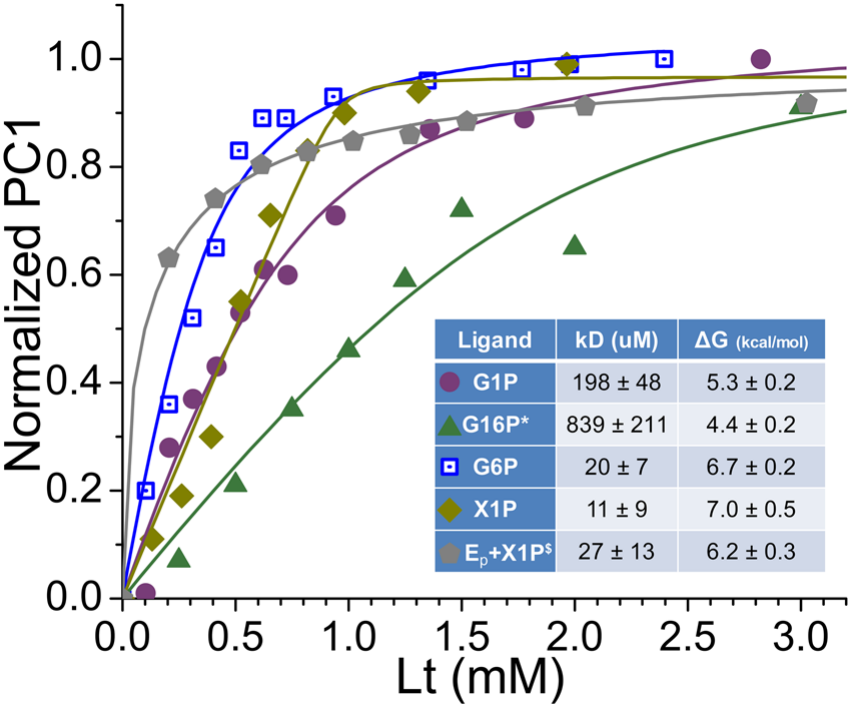
Binding isotherms and affinities of PMM/PGM(S108C) for phosphosugars derived from ^15^N TROSY NMR spectra. The binding isotherms were obtained using PCA implemented with TREND software^16, 35^. PMM/PGM(S108C) was titrated with G1P (purple circles), G6P (blue squares), G16P (green triangles), or X1P (yellow-green diamonds). Fitted dissociation constants and Gibbs free energies are tabulated in the inset. The X1P titration of wt enzyme (gray pentagons) was collected at 298 K in the slow exchange regime and is adapted from ref. 16.

Binding isotherms were obtained from principal component 1 (PC1) from PCA of comprehensive lists of ^15^N TROSY peaks from titrations^16^ (Fig. 3). Ligand binding affinities were obtained by fitting binding isotherms to Eq. 3. Apparent dissociation constants of G1P, G6P, and X1P from the S108C-inactivated enzyme are 198 ± 48, 20 ± 7, and 11 ± 9 μM, respectively (Fig. 3). However, the affinity of G16P for PMM/PGM(S108C), with *K*_*D*_ of 839 ± 221 μM, seems to be impaired by the S108C lesion relative to apparent G16P affinity for wt enzyme, which appears to be at least an order of magnitude greater (Supplementary Fig. S4C). X1P binding to wt PMM/PGM at 308K is accompanied by peaks undergoing fast, intermediate, or slow exchange, complicating derivation of a binding isotherm from the peak lists. Consequently, we obtained the affinity for X1P by applying PCA directly to spectra of an X1P titration at 298 K^16^, where its amide peaks are in the slow exchange regime (Fig. 3A). Both wt and PMM/PGM(S108C) display the most affinity for the X1P inhibitor.

### Clustering of ligand binding responses

**Figure 4:**
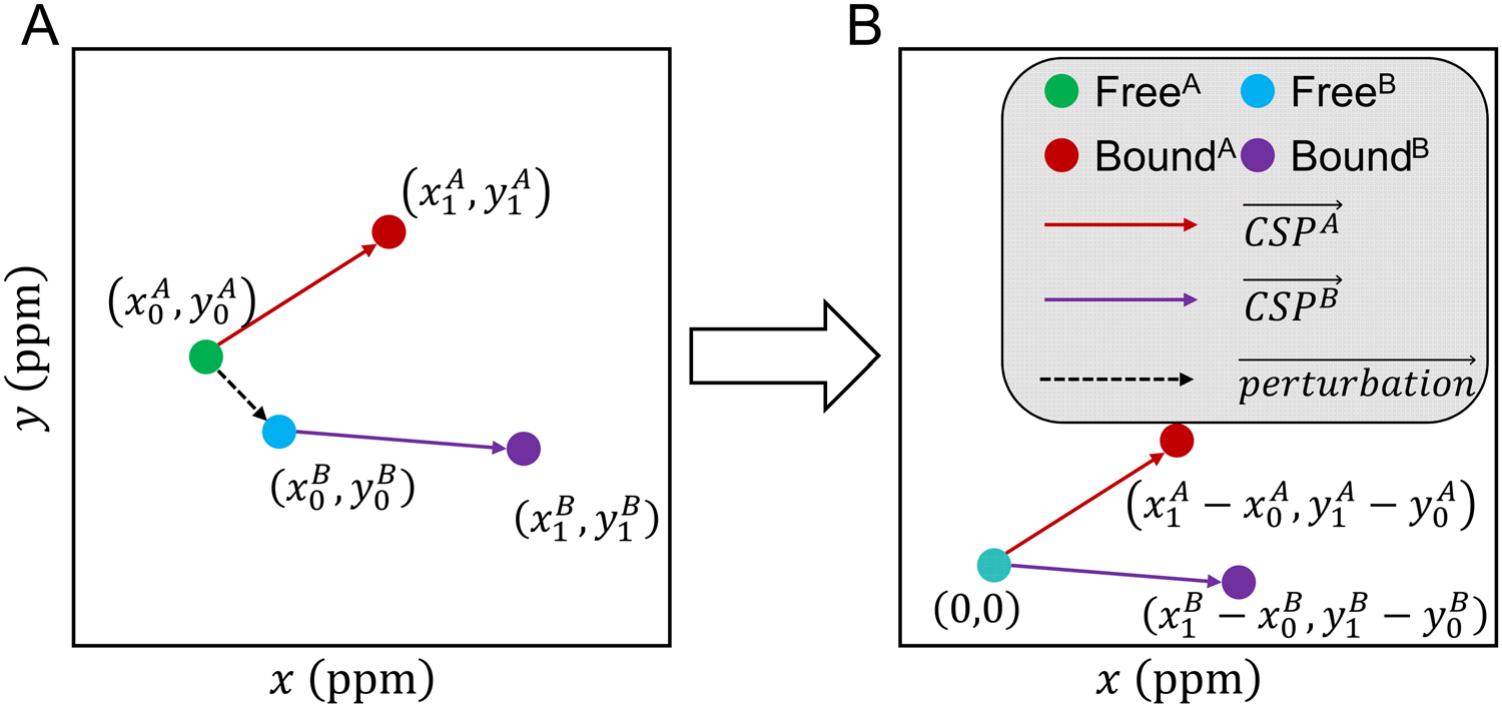
Calculation of CSP vectors to represent peak shifts from ligand binding for the PCA and CONCISE analyses. The x-axis refers to the ^1^H chemical shift scale of the TROSY spectra and the y-axis the ^15^N chemical shift scale normalized by 0.2-fold to the ^1^H scale. (**A** and **B**) refer to wt PMM/PGM and PMM/PGM(S108C). (**A**) Green and cyan symbols represent their initial peak positions and brown and purple symbols their positions when shifted by ligand or covalent modification. (**B**) In order to compare perturbations of covalently different A and B species, their initial peak positions are shifted to the origin at (0, 0). The CSP vectors of A and B share this origin. Ligand-binding perturbations refer to the initial free state as the origin. E_deP_ refers to E_P_ as the origin. In the biplots, the S108C perturbation refers to wt E_P_ as the origin.

**Figure 5:**
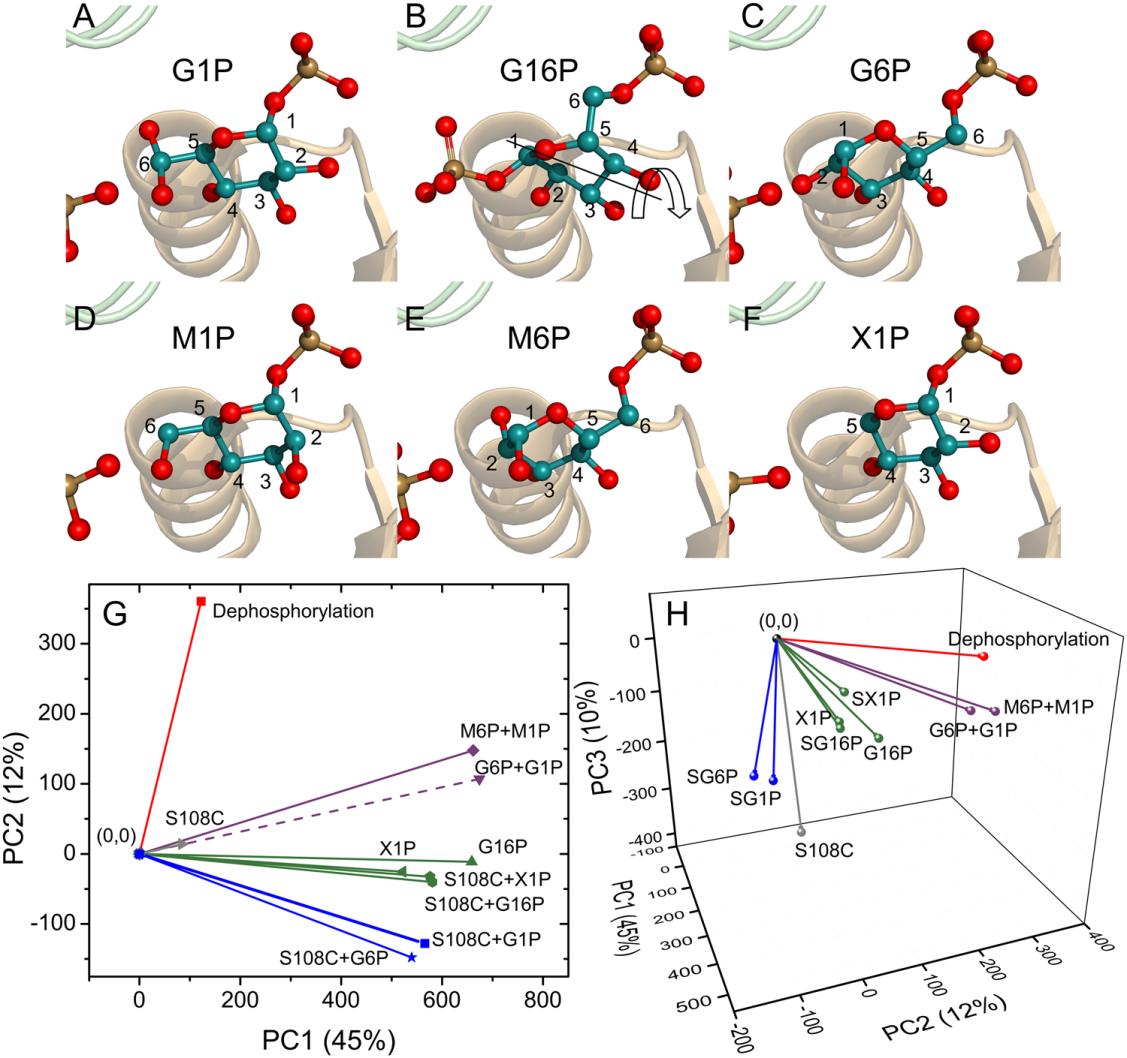
Clustering and resolution of ligand binding and other perturbations of PMM/PGM. The active site region of crystallographic structures is plotted for complexes with (**A**) G1P, (**B**) G16P, (**C**) G6P, (**D**) M1P, (**E**) M6P, and (**F**) X1P. The PDB accession codes are (**A**) 1P5D, (**B**) 2FKM, (**C**) 1P5G, (**D**) 1PCJ, (**E**) 1PCM, and (**F**) 2H5A. The arrow in (**B**) symbolizes the 180° rotation of G16P. (**G**) The PCA biplot of PC1 and PC2. See Fig. 4 for the definition of the CSP vectors used in monitoring the perturbations. The colors of the vectors point out the results of K-means clustering of PC1-2. (H) The PCA biplot of PC1, PC2, and PC3 is colored with K-means clustering using PC1-3.

The spectral perturbations by ligands, phosphorylation, or mutation were analyzed by PCA implemented with singular value decomposition (SVD) in a manner similar to CHESCA^18^. The PCA analyses of the spectra should be regarded as *qualitative* comparisons, because in some cases the enzyme was saturated by a mixture of ligands. Wt PMM/PGM transformation of 10 µM G1P reaches a steady state with a mixture of approximately 70% G6P, 20 to 25% G1P, and 5 to 10% G16P^8^, but with substrate inhibition (*K*_*i*_ = 110 μM) at higher concentrations^23^. The NMR peaks probably often represent a weighted average of the complexes present because rapid off-rates and fast chemical exchange accompany the range of affinities. In order to compare perturbations of wt and S108C mutant enzymes, we shifted the reference peak positions to a shared origin at (0, 0) and refer to the peak shifts as CSP vectors (Fig. 4). We applied PCA to the CSP vectors of 183 residues with TROSY NMR peaks identified under all 11 conditions of the wt and mutant enzyme compared. We plotted the results as biplots for separation and clustering^24^. The similarity among the spectra perturbed by the ligands (Supplementary Figs S1–S3) is suggested by five clusters present in biplots of the first two or three PCs (Fig. 5G,H). While PCs 1 to 3 account for only 67% of the variance (Supplementary Fig. S5), they suffice to define meaningful clusters. (Adding PC4 only moves the X1P complex to a less likely cluster). Dephosphorylated wt enzyme (E_deP_) and PMM/PGM(S108C) each map to distinct clusters (Fig. 5G). Although the S108C mutation removes the phosphorylation at this site, its vector is nearly perpendicular to the vector for E_deP_ (Fig. 5G,H), suggesting independent behavior of these two perturbations. Three clusters for phosphosugar binding are evident in the biplots (Fig. 5G,H). The NMR spectral responses to G6P and mannose 6-phosphate (M6P) form one cluster in binding wt PMM/PGM and another cluster when binding PMM/PGM(S108C). This suggests that pSer108 in the active site influences the spectral response to substrates. Binding of G16P and X1P to wt PMM/PGM and PMM/PGM(S108C) belong to another cluster, despite their structural and electrostatic differences. This cluster might suggest that S108C does not affect association with G16P and X1P as much. However, the S108C lesion seems to impair affinity for G16P (c.f. Fig. 3 and Supplementary Fig. S4C). This mutation introduces disorder to this key loop in the active site in the crystal structure^22^, possibly impairing association of G16P, which normally supplies the phosphoryl group for transfer to Ser108. S108C-altered binding of G16P is also suggested by distinctive shifts to NMR peaks of the S108C mutant for residues 250, 258, 320, and 328 near the active site (c.f. Fig. 2A and Supplementary Fig. 4A). Comparison by PCA biplots has discerned differences among these perturbations.

### Free and X1P-bound states define extremes of conformational equilibria

**Figure 6:**
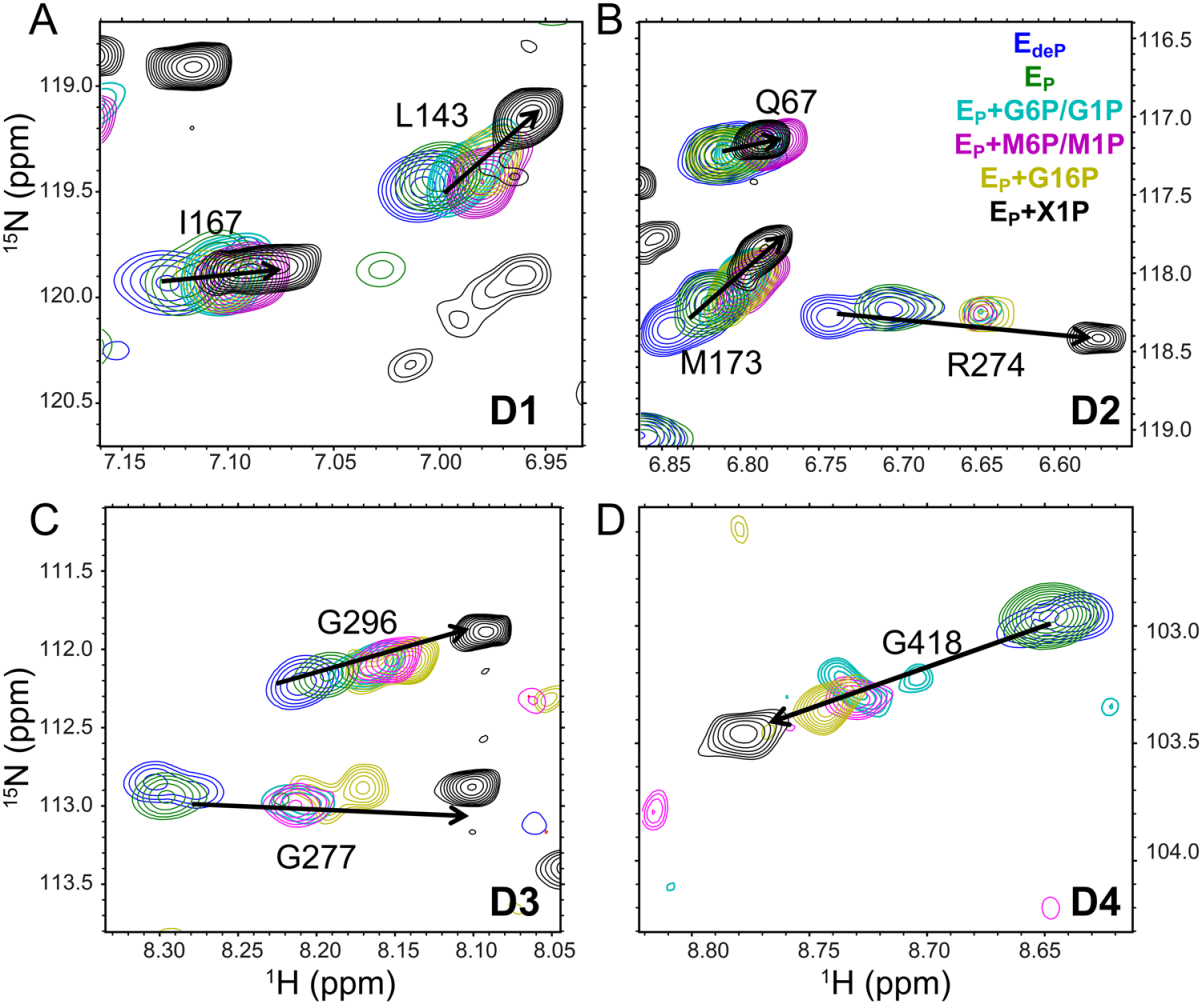
Examples of concerted chemical shift changes in phosphosugar titrations of wt PMM/PGM observed by ^15^N TROSY at 800 MHz. Examples in domains 1, 2, 3, and 4 are shown in panels (**A**,**B**,**C** and **D**), respectively. Blue contours represent E_deP_ (apo), green for E_P_ (apo), cyan for E_P_+G6P with G1P, purple for E_P_+M6P with M1P, yellow for E_P_+G16P, and black for E_P_+X1P.

To compare the averaged conformational equilibria of PMM/PGM with ligands or mixtures of ligands present, we applied a chemical shift-based statistical approach. COordiNated Chemical Shifts bEhavior (CONCISE) evaluates the relative size and statistical distribution of chemical shift changes in trajectories of amide NMR peaks. In contrast to other PCA analyses of chemical shifts^13, 15, 16, 18^, CONCISE applies PCA to the shifting peak of each residue individually and retains only its PC1. This PC1 captures the largest variance for that residue, which suggests the residue sensing of relative equilibrium positioning^21^. CONCISE assesses the “density of probabilities” of each ensemble of conformations^21^. Examples of amide peaks from each domain with a large PC1 component are shown in Fig. 6. “Population distributions” for each set of conditions of the enzyme were estimated from their projections onto the PC1 direction (marked by black arrows in Fig. 6).

**Figure 7:**
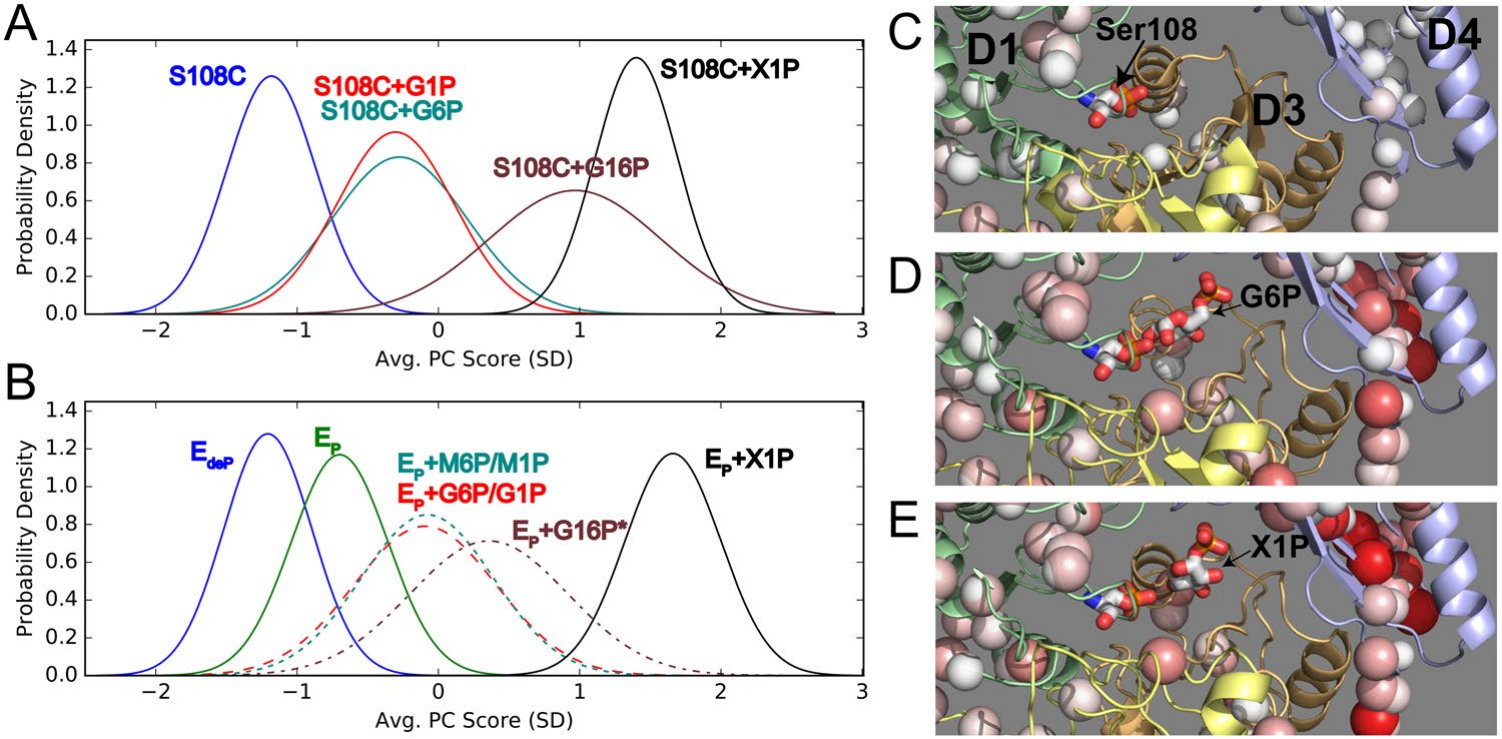
CONCISE analysis of PMM/PGM complexes and mixtures. (**A**,**B**) Probability densities of different states are plotted in units of standard deviations of average PC scores for (**A**) PMM/PGM(S108C) and (**B**) wt PMM/PGM. The dashed curves in (**B**) symbolize mixtures of phosphosugars present with the minor phosphosugar listed second. * refers to the likelihood of the G16P added to wt enzyme being transformed in part to G6P and G1P. (**C**–**E**) Generally increasing amide CSPs accompany the progression of increasing average PC scores. The CSPs are calculated relative to E_P_ using Eq. 1. Increasing CSPs of (**B**) E_deP_, (**C**) E_P_+G6P, G1P (major, minor sugars), and (**D**) E_P_+X1P states are symbolized by increased radius and red tint of the spheres plotted on the crystal structures with PDB accession codes of 1K35, 1P5G, and 2H5A, respectively.

For CONCISE analysis, PMM/PGM(S108C) has the advantage of limited catalytic turnover, which extends the lifetime of the G1P substrate to days^22^. CONCISE identified 83 linearly shifting amide NMR peaks distributed across all four domains of the mutant enzyme (Supplementary Fig. S6A). The free state and complex with X1P occupy the extremes of the continuum (Fig. 7A). The populations with G1P or G6P are overlapped at an intermediate position (Fig. 7A). The population density for the PMM/PGM(S108C) complex with G16P is wide and closer to that of the X1P complex. CONCISE of the wt enzyme used 89 residues with linear amide chemical shift trajectories from the perturbations (Supplementary Fig. S6B). The size of the CSPs generally increases with these scores (Fig. 7C–E). The dephosphorylated free (E_deP_) state defines the most open form of the enzyme. The population density distribution for G6P averaged with a smaller amount of G1P produced from it overlaps that of M6P averaged with a lesser amount of M1P. These two peaks occupy an intermediate position similar to that of the more homogeneous complexes with PMM/PGM(S108C) (Fig. 7A,B). Next to them reside the broad population for E_P_ with G16P in a presumed mixture of with G6P and G1P. The E_P_ complex with the X1P inhibitor lies at the closed extreme (Fig. 7B).

### Slowing of hydrogen exchange by association with X1P inhibitor

**Figure 8:**
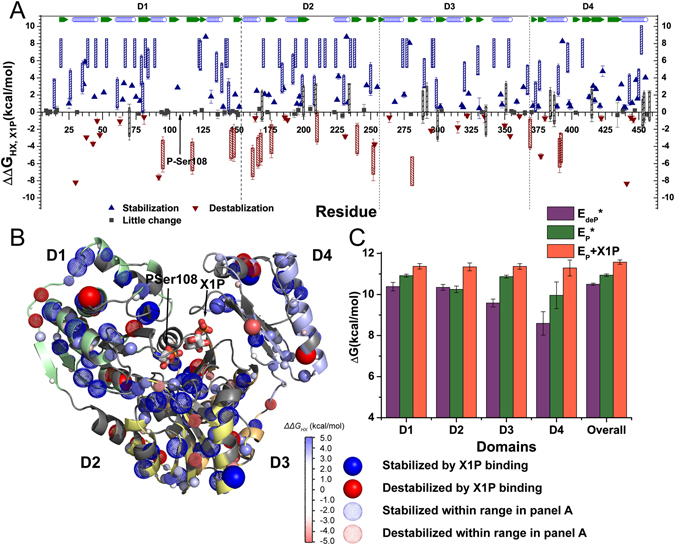
X1P binding protects more sites from hydrogen exchange than it exposes. (**A**) Differences of free energies of hydrogen exchange (*ΔΔG*_*HX*_, _*X1P*_) subtract the free energies for E_P_ from those of E_P_+X1P. Triangles indicate cases where *k*_*ex*_ was measured in both E_P_ and E_P_+X1P forms. Hatched bars represent cases in which *k*_*ex*_ in one state is unmeasurable and estimated to lie in the range 1 s^−1^ > *k*_*ex*_ > 4 × 10^−3^ s^−1^. (**B**) The *ΔΔG*_*HX*_, _*X1P*_ values are mapped onto the crystal structure of E_P_+X1P (PDB:2H5A). Amide groups protected from HX by X1P binding are marked by blue spheres. Amide groups destabilized (mobilized) by X1P binding have red spheres. Dotted spheres correspond to residues with uncertainty ranges marked as hatched bars in (**A**). The magnitudes of *ΔΔG*_*HX*_, _*X1P*_ are symbolized by the color gradient, as well as by the radii of the spheres. (**C**) Estimated folding stabilities are plotted for each domain and the enzyme as a whole, not only for E_P_+X1P from this study, but also for E_P_ and E_deP_ compared in ref. 10.

The effects of inhibitor binding on the stability and flexibility of wt PMM/PGM were evaluated by comparing the hydrogen exchange (HX) behavior in the presence and absence of X1P. Residue-specific free energies of HX protection were inferred from measurements of E_P_+X1P relative to the free energies reported for the phosphorylated free state E_P_^10^. Conditions of pH 7.4 and 35 °C ensured that the HX behavior of the X1P complex occurred in the bimolecular EX2 regime, where the residues with the largest *ΔG*_*HX*_ can be used to estimate folding stability by the method of ref. 25. Both subsecond HX and slow hydrogen-deuterium exchange (HDX) of E_P_+X1P were measured, revealing rate constants and *ΔG*_*HX*_ for 201 amide groups. The *ΔG*_*HX*_ values of E_P_+X1P are plotted together with those of E_P_ for comparison (Fig. S7). Seventy-four amide groups in E_P_−X1P decay too slowly to be quantified by HDX-NMR. Consequently, lower bounds on their exchange rates were set to *k*_*ex*_ of 5.56 × 10^−5^ min^−1^, which is the slowest *k*_*ex*_ value that could be measured for E_P_ and E_deP_^10^. Only 20 groups of E_P_ required this estimate. *ΔΔG*_*HX*_ values calculated as differences between *ΔG*_*HX*_ of E_P_−X1P and E_P_ are plotted in Fig. 8A and mapped onto the crystal structure (Fig. 8B). Amide groups stabilized by X1P binding are interspersed throughout all four domains. Residues distant from bound X1P are stabilized as much as those nearby, implicating long-range effects of the association with this ligand.

Although the overall effect of X1P is stabilizing, 25 residues located at least 10 Å from the binding site appear to be destabilized (*ΔΔG*_*HX*_ < 2 kcal/mol), i.e. with accessibility to HX increased by the X1P bound (red spheres in Fig. 8B). Proteins that undergo a mixture of slowing, acceleration, and no change of HX upon ligand binding have been attributed to a mixture of effects on the free energies of the excited states, which are competent for HX^26^. This can account for the mix of hydrogen bonds being stabilized, destabilized, or unaffected by ligand binding that is stabilizing to the ground state^26^. Estimations of global folding stability using the amide groups most protected from HX^25^ are not, however, affected by localized accelerations of HX. The folding stability estimated for the entire enzyme suggests that the X1P-bound state is globally stabilized by ~0.6 kcal/mol over the E_P_ state. The estimates for each individual domain show that all four domains rise to a uniform level of stability, with the stability of D2 and D4 increasing the most (Fig. 8C).

## Discussion

### Five enzyme states resolved by CONCISE analysis of NMR chemical shifts

Crystallographic characterizations of PMM/PGM from *P. aeruginosa* ^6, 27–29^ have mainly observed a ligand-closed conformation or an open, free state. Mechanistic enzymology indicates that the bisphosphorylated intermediate G16P must rotate by 180° to complete the catalytic cycle^7, 8^. This rotation implies the loss of active site contacts with G16P, which could occur by partial opening of the cleft^8^ and/or by enough mobility of the catalytic pocket for rotation of G16P to proceed. One crystal structure with the cleft half-open suggested partial opening also^11^. Subsequent comparisons of the free states *in solution* recognized E_deP_ to be more open and flexible and E_P_ to be more closed and stable due to the phosphorylation-induced electrostriction of the catalytic cleft^9, 10^. Together, these studies suggested fluctuations of the cleft from open, partially open, to closed.

CONCISE and PCA comparisons of NMR spectra under various conditions each reveal a more complex conformational landscape than was evident previously. These approaches resolve the E_deP_ and E_P_ free states of wt PMM/PGM. They also resolve the G16P complex with PMM/PGM(S108C) in solution (Figs 5G,H and 7A). Importantly, the scores from the CONCISE analysis of phosphosugar complexes with PMM/PGM(S108C) suggest three discernible states: (i) monophosphosugar complexes, (ii) a bisphosphosugar population and (iii) the X1P inhibitor complex (Fig. 7A). Thus, five states of the enzyme are now recognizable. What might the different positioning of the ligand complexes on the continuum mean? One possibility is that the three types of complexes differ in the degree of dynamic averaging of the cleft opening. The higher affinity X1P complex may have the highest probability of closure of the cleft. The broad distribution of the population density of the G16P complex (Fig. 7A) suggests it is heterogeneous, encompassing multiple substates. Additional peaks in TROSY spectra of E_P_+G16P (Supplementary Fig. S2) may also be consistent with a mixture of states. Addition of G16P to wt PMM/PGM must result in formation of G6P and G1P. Catalytic cycling is also likely between the two orientations that G16P can adopt in the active site (Fig. 1B). The similar breadth of the CONCISE population densities for the G16P mixtures with wt or S108C-impaired PMM/PGM suggest that some of the heterogeneity of substates persists through changes in activity and phosphorylation.

### PCA clustering corroborates and complements the view of multiple states

The PCA biplots distinguish the free states E_P_ and E_deP_ from each other and from the ligand-bound states (Fig. 5G,H), though crystallography does not. The biplots cluster the complexes with singly phosphorylated substrates (Fig. 5G,H), suggesting their conformational similarity, as did crystallography^6^. Binding of X1P and G16P to wt PMM/PGM or the S108C mutant converges to a different cluster (Fig. 5G,H), implying their distinction from the monophosphosugar substrate complexes. CONCISE results also suggest this difference. By contrast, crystallography has not been able to differentiate these among the closed conformations of these complexes. Overall, the PCA clusters confirm and resolve the major equilibrium states similarly to CONCISE with resolution of the E_P_ and E_deP_ free states, recognition of the shared behavior of monophosphosugar substrate complexes, and differentiation of them from the G16P and X1P complexes (Figs 5G, H and 7).

### Implications of dephosphorylated PMM/PGM(S108C) for ligand binding

Despite the S108C lesion introducing crystallographic disorder to the phosphoserine loop in the active site^22^, this mutation maintains similar to wild-type positioning of the states identified by CONCISE, but without a phosphorylated state (Fig. 7A,B). Thus, it appears that phosphorylation of Ser108 does not change the pattern of conformational transitions in response to binding of phosphosugars. This suggests that phosphorylation of Ser108 may not play an important role in the global response of PMM/PGM to phosphosugar binding.

The PCA biplots distinguish between the wt and S108C-substituted free states and their complexes with substrates G6P and M6P (Fig. 5G,H). This implies that the S108C mutation has consequences beyond dephosphorylation, consistent with the loss of electron density in the loop in the active site observed crystallographically^22^. The convergence in the PCA biplots of the wt and S108C cases of the X1P complex and G16P mixtures suggests that these ligands might partially overcome the disturbance of the loop by the S108C mutation.

Although phosphorylation of Ser108 compacts and stabilizes PMM/PGM by introducing electrostatic attraction between domains 3 and 4^9, 10^, the removal of this phosphorylation by the S108C lesion has little effect on affinity for X1P (Fig. 3). This supports the proposal that the attraction of the substrate’s phosphoryl group for domain 4 is a key determinant of ligand-binding affinity^6^.

### Affinity and stability correlations with progression of enzyme states

The CONCISE approach ranks the enzyme states by PC scores as: X1P complexes > G16P mixtures > G6P, M6P mixtures > free E_P_ > free E_deP_ (Fig. 7A,B), qualitatively correlating with the relative affinities for X1P > G6P > G1P (Fig. 3). This could be consistent with a greater probability of ligand binding being related to a greater shift in the lifetime of the ligand-closed state of PMM/PGM. This would be analogous to the progression between closed and open states (ternary-binary-apo) reported in the seminal CONCISE analysis of a domain of protein kinase A^21^. The global protein folding stabilities, from NMR-detected hydrogen exchange, may follow this same general progression with E_P_+X1P > E_P_ > E_deP_ (Fig. 8C). Association with X1P promotes uniform folding stability across the enzyme, suggesting that X1P bridges the four domains together. In contrast, transient closing of the cleft by phosphorylation^10^ is not as stabilizing of the independent domain 4 (Fig. 8C). The X1P complex possessing the highest average PC score from CONCISE potentially correlates with greater rigidity. Thus, phosphorylation and binding of phosphosugars appear to shift the equilibrium progressively from the more open, flexible and less stable dephosphorylated state to a more closed, rigid and stabilized phosphorylated state.

## Conclusions

Qualitative PCA and CONCISE comparisons of NMR-monitored ligand titrations suggest three ligand-closed states and corroborate distinct E_deP_ and E_P_ free states. This strongly suggests that the conformational and functional landscape of PMM/PGM is more varied in solution than in crystals. Domain 4 appears more important than catalytic Ser108 for the enzyme’s collective responses to phosphosugars. The effects of ligand-binding are greatest at the interface between domains 3 and 4, but are global and long-range nonetheless, based on both NMR peak shifts and hydrogen exchange protection. Phosphorylation and phosphosugar binding progressively shift PMM/PGM along a continuum from the dephospho-enzyme towards the inhibited complex. This study has resolved additional states in a representative enzyme from the ubiquitous α-D-phosphohexomutase superfamily. PCA and CONCISE comparisons of NMR spectra appear promising for characterizing multiple collective responses of enzymes and proteins to ligand binding and covalent modifications.

## Methods

### NMR spectroscopy

Isotope-labeled samples for NMR acquisition were prepared with protein concentrations of 0.5 to 1.0 mM in 50 mM MOPS (pH 7.4), 1 mM MgCl_2_ with 5% D_2_O (v/v). Phosphosugars were titrated into ^2^H/^15^N PMM/PGM(S108C) at 310 K or wt PMM/PGM at 308 K on a Bruker Avance III 800 MHz spectrometer with TCI cryoprobe. A series of 2D ^15^N BEST-TROSY spectra^30^ were collected with phosphosugar additions up to 8-fold molar excess.

HX was measured with a 5-fold molar excess of X1P. HX transpiring in milliseconds was measured with improvements to a CLEANEX-PM pulse sequence^31, 32^ and fitting described before^10^ and in Supporting Material. HDX occurring in hours was measured and fitted as described^10^ and in Supporting Material.

### Interpretation of NMR spectra

The spectra were processed with NMRPipe^33^ and analyzed using Sparky^34^. The peaks were interpreted with the chemical shift assignments reported^22^. Chemical shift perturbations (CSPs) of amide peaks were calculated as the radius:

1 $${\rm{\Delta }}{\delta }_{NH}=\sqrt{({(\frac{{\rm{\Delta }}{\delta }_{N}}{5})}^{2}+{\rm{\Delta }}{{\delta }_{H}}^{2})}$$

where $\delta N$ and $\delta H$ are the changes in ^15^N and ^1^H dimensions in units of ppm. The ^15^N frequency changes were scaled down by a factor of 5 to normalize them to the ^1^H scale. Each TROSY spectrum was referenced for agreement with the reference spectrum of free E_P_ by minimizing the root-mean-square deviation of the 60 peaks with the smallest CSPs between the two spectra:

2 $$RMSD=\sqrt{{\sum }_{i=1}^{{N}_{res}}({(\frac{{\delta }_{Ni}-{\delta }_{Ni}^{ref}}{5})}^{2}+{({\delta }_{Hi}-{\delta }_{Hi}^{ref})}^{2})}$$

The reconciling shifts averaged 0.0014 ± 0.0034 ppm for the ^1^H axis and 0.0023 ± 0.0044 ppm for the ^15^N axis.

### Measurements of affinity

Binding isotherms of all titrations were calculated by applying PCA to lists of the peaks picked from each of the spectra of the titration^16^ using the program named TREND^35^. Each binding isotherm was fitted to Eq. 3

3 $$p{}_{bound}=\Vert PC1\Vert =\frac{({[P]}_{t}+{[L]}_{t}+{K}_{D})-\sqrt{{({[P]}_{t}+{[L]}_{t}+{K}_{D})}^{2}-4{[P]}_{t}{[L]}_{t}}}{2{[P]}_{t}}$$

where *p*_*bound*_ is the fraction of protein (of total concentration *[P]*_*t*_) bound to ligand. [L]_t_ is the total ligand concentration. ||PC1|| is the normalized principal component 1 obtained by TREND, and indicates the change in the population of the bound state^16, 35^. The estimates of uncertainties of *K*_*D*_ are the fitting uncertainties from nonlinear least squares fits of Eq. 3 to PC1 using OriginPro. This uncertainty was normalized by *K*_*D*_ in propagating the error to the free energy of association.

### Vectors for shifts of NMR peaks

A CSP vector tracks the shift of a peak from its position in the free state to its position after near-saturating addition of ligand. The vector is calculated as the difference between the peak coordinates: $({x}_{end}-{x}_{ini},\,{y}_{end}-{y}_{ini})$ $({x}_{end}-{x}_{ini},{y}_{end}-{y}_{ini})$. Examples of calculating CSP vectors for PMM/PGM are shown in Fig. 4. These vectors focus on the change in perturbed peak position and remove the initial differences in peak positions of free states of wt and mutant enzymes, in order to include wt and mutant spectra in the same PCA biplot comparison. All shifts of NMR peaks from the peak lists from the titrations were converted into CSP vectors.

### PCA clustering of spectra perturbed by ligand, dephosphorylation, or mutation

PCA biplots were calculated with the TREND software package^35^ on CSP vectors for each set of ligand-induced spectral changes. The software converted the lists of CSP vectors were converted into a 2D matrix^16^, with each column representing a list of CSP vectors. The ^1^H and ^15^N coordinates alternate in the column list. The columns of the input matrix were centered and autoscaled^18^. Clustering of the perturbed spectra was judged from biplots of PCs 1 to 3 using the K-means clustering algorithm^36^ implemented using the scikit-learn package (http://scikit-learn.org/).

### CONCISE analysis

COordiNate ChemIcal Shift bEhavior (CONCISE) was used to monitor linear trajectories of CSP vectors and measure the equilibrium position of each free or ligand-bound state^21^. The direction of largest covariance of the peaks of each residue was analyzed independently by PCA. Standard deviations (SD) of PC1 and PC2 were used to calculate linearity of the peak positions from all states for each residue. Residues with poor linearity (SD_PC1_/SD_PC2_ < 3.0) and/or with small perturbations (PC1 < 0.05 ppm) were discarded from the analysis to reduce systematic error^21^.

## Electronic supplementary material

Supplementary Information
