# Supplementary material for: Multiple Ligand-Bound States of a Phosphohexomutase Revealed by Principal Component Analysis of NMR Peak Shifts
Source: Sci Rep. 2017 Jul 13;7:5343. doi: 10.1038/s41598-017-05557-w (PMC5509744; doi:10.1038/s41598-017-05557-w)
Supplement: Supplementary file 1 — Supplementary Information [file 41598_2017_5557_MOESM1_ESM.doc]

Supplementary Information for:

Multiple Ligand-Bound States of a Phosphohexomutase Revealed by Principal Component Analysis of NMR Peak Shifts

Jia Xu, Akella V. S. Sarma, Yirui Wei, Lesa J. Beamer, and Steven R. Van Doren*

Biochemistry Dept., 117 Schweitzer Hall, University of Missouri, Columbia, MO 65211 USA

* email: [vandorens@missouri.edu](mailto:vandorens@missouri.edu)

**Supplementary Methods**

**Enzyme preparation and isotopic labeling**. ^2^H, ^15^N labeled His-tagged PMM/PGM and its S108C mutants were expressed and purified according to the protocol available^1,2^. The phosphorylation states of ^2^H/^15^N labeled PMM/PGM samples used for G6P, G16P, M6P, and X1P titrations were estimated to be initially75 ± 8%, 77 ± 9%, 77 ± 10%, and 66 ± 12%, respectively, and rising to > 80% phosphorylation after half saturation with the ligands. This assessment used the well-resolved peaks of the phosphorylated (E_p_) and dephosphorylated (E_dep_) forms of the enzyme as described^3^. (Time courses for the dephosphorylation and effects of phosphosugars to increase phosphorylation have been reported^3-5^). Xylose 1-phosphate was synthesized by Prof. Thomas Mawhinney (University of Missouri).

**Hydrogen exchange analysis**. The rates of rapid hydrogen exchange (HX) of amide groups were detected by TROSY-detected CLEANEX-PM NMR pulse sequence, acquired with interleaved acquisition of the mixing times^3,6^. The hydrogen exchange rates on the rapid time scale were obtained by fitting to:

$\frac{I}{I_{0}}=\frac{k_{ex}}{R_{1A}+k_{ex}-R_{1B}}\times[\exp(-R{}_{1B}\tau_{m})-\exp[-(R_{1A}+k_{ex})\tau_{m}]]$ Eq. S1

where I/I_0_ stands for normalized peak heights, *R_1A_* is a combination of transverse and longitudinal relaxation, *k* is the amide HX rate constant, and *R_1B_* is the water relaxation constant during the mixing time. Since *R_1B_* values ranging from 0.1 to 0.001 s^-1^ make little difference to the fitted *k_ex_* values, 0.01 s^-1^ was used^7^.

HX rates of slowly exchanging amides were characterized according to protocols detailed in ref ^3^. H_2_O-based buffer dissolved ^2^H/^15^N samples were concentrated to ~5 mM and the diluted to ~1 mM in D_2_O-based buffer at room temperature. Exponential decay rate constants in D_2_O (*k_obs_*) were obtained by fitting peak heights to a single exponential function. Due to turbidity developing over time, hydrogen-deuterium exchange (HDX) rates (*k_ex_*) were obtained by subtracting by subtracting decay rates measured in a control NMR experiment using H_2_O instead of D_2_O: *k_ex_*= *k_obs_*- *k_control_*.

Experimental protection factors of amide hydrogens were calculated as free energy differences between the measured folded state and the random coil reference state:

$\Delta G=-RT\ln(\frac{k_{ex}}{k_{rc}})$ Eq. S2

Estimates of intrinsic rate constants of random coil HX (*k_rc_*) were obtained using the SPHERE server (<http://www.fccc.edu/research/labs/roder/sphere/sphere.html> ).

**Table S1. Assigned peaks of ^15^N TROSY NMR spectra shown in Figures S1 to S3**.

|  | | E_deP_^¶^ | E_p_^¶^ | | | | E_p_+  G6P/G1P | | | E_p_+G16P^‡^ | | E_p_+  M6P/G1P | | | E_p_+X1P | | |  | | | | |
| --- | --- | --- | --- | --- | --- | --- | --- | --- | --- | --- | --- | --- | --- | --- | --- | --- | --- | --- | --- | --- | --- | --- |
| Assigned | | 382 | 446 | | | | 354 | | | 333 | | 341 | | | 409 | | |  | | | | |
| Unassigned | | 64 | 0 | | | | 92 | | | 113 | | 105 | | | 37 | | |  | | | | |
| CSP > 1σ * | | 56 | 0 | | | | 54 | | | 44 | | 44 | | | 47 | | |  | | | | |
| CSP > 2σ * | | 23 | 0 | | | | 15 | | | 15 | | 16 | | | 17 | | |  | | | | |
|  |  | | |  | | | |  | | |  | | |  | | |  | | |  |  | |
|  | | S108C^♯^ | | |  | S108C  +G1P | | | S108C  +G16P | | | | S108C  +G6P | | | S108C  +X1P | | |  | | |  |
| Assigned | | 484 | | |  | 471 | | | 423 | | | | 458 | | | 395 | | |  | | |  |
| Unassigned | | 0 | | |  | 13 | | | 61 | | | | 26 | | | 89 | | |  | | |  |
| CSP > 1σ ^$^ | | 0 | | |  | 56 | | | 64 | | | | 63 | | | 49 | | |  | | |  |
| CSP > 2σ ^$^ | | 0 | | |  | 18 | | | 23 | | | | 24 | | | 19 | | |  | | |  |

* Compared with E_p_

^¶^ Peak assignments reported^1^ and available under entry 17602 at the BioMagResBank

^‡^ E_P_+G16P also generates G1P and G6P

^#^ Peak assignments reported^1^ and available under entry 17652 at the BioMagResBank

^$^ Compared with S108C


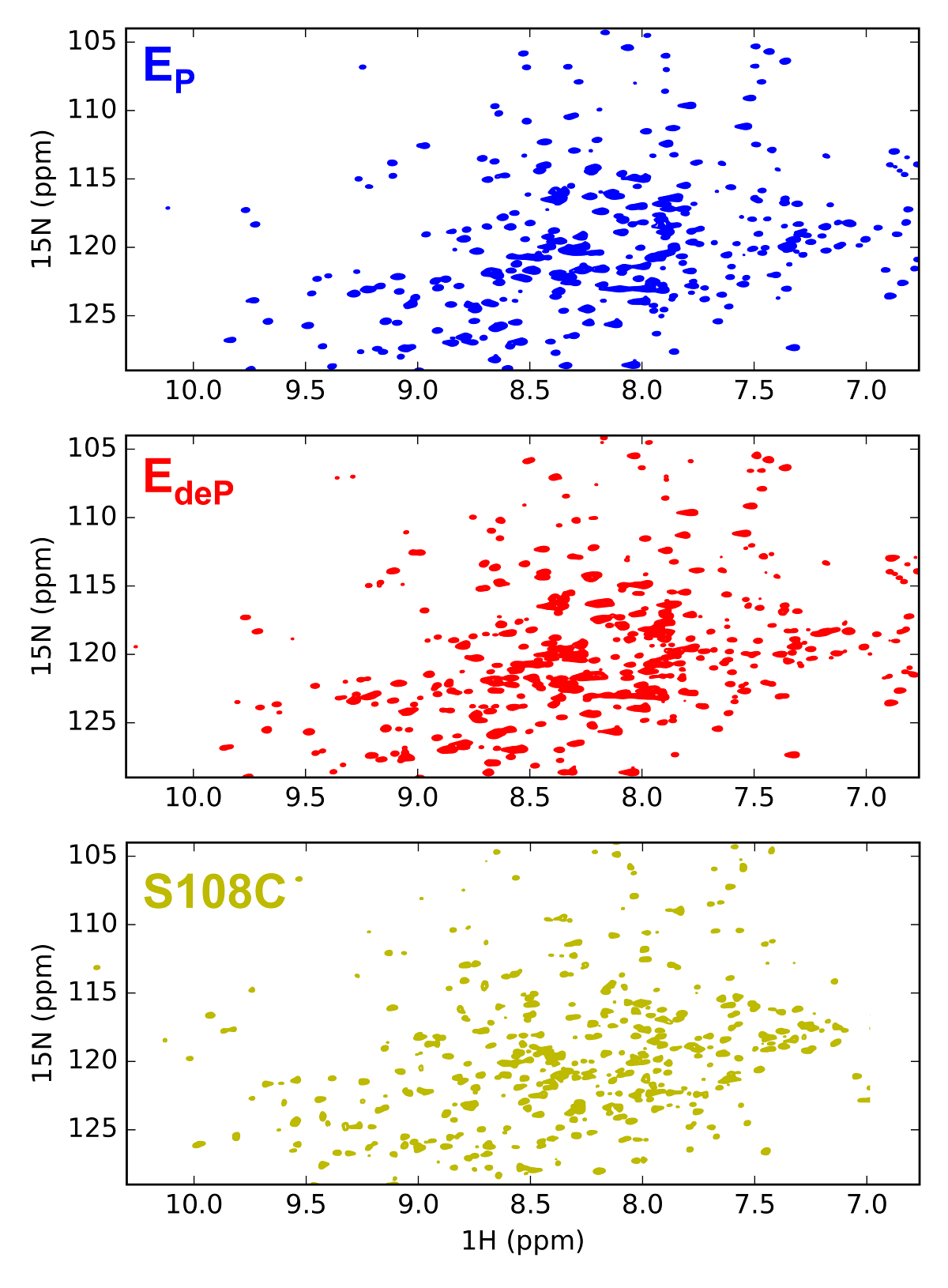


**Figure S1. ^15^N TROSY spectra of the free states of PMM/PGM from *Pseudomonas aeruginosa*.** E_P_ (blue) represents the wild-type with phosphorylation, E_deP_ (red) the wild-type without phosphorylation, and S108C (yellow) the mutant with catalysis suppressed. The spectra were collected at pH 7.4 on a Bruker Avance III 800 MHz spectrometer with TCI cryoprobe.


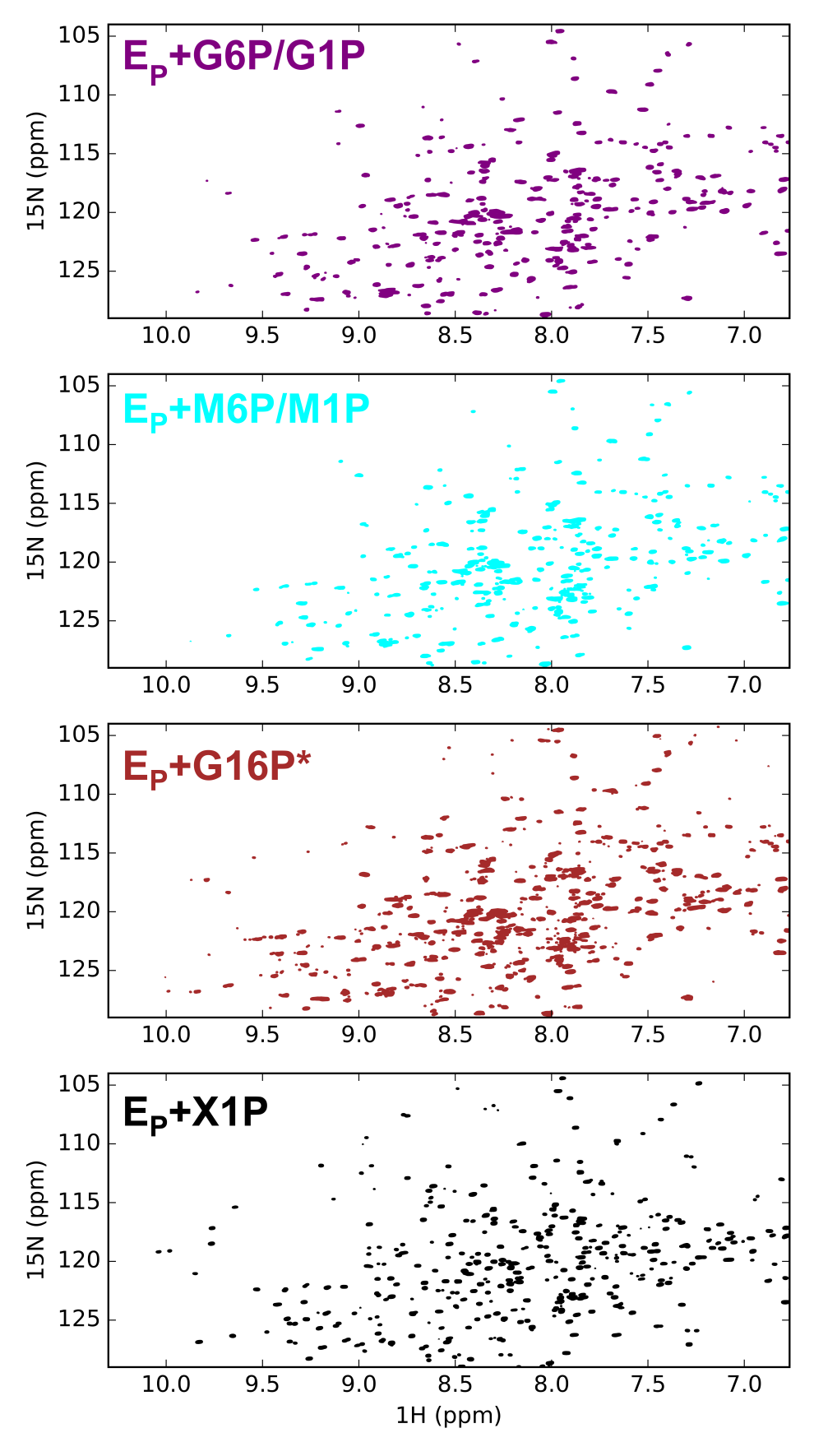


**Figure S2. ^15^N TROSY spectra of E_P_ at 308 K with additions of G6P (purple), M6P (cyan), G16P (brown), or X1P (black).** Also listed is the phosphosugar produced, but at lower concentration than the added substrate remaining. * E_P_+G16P also generates G1P and G6P.

**
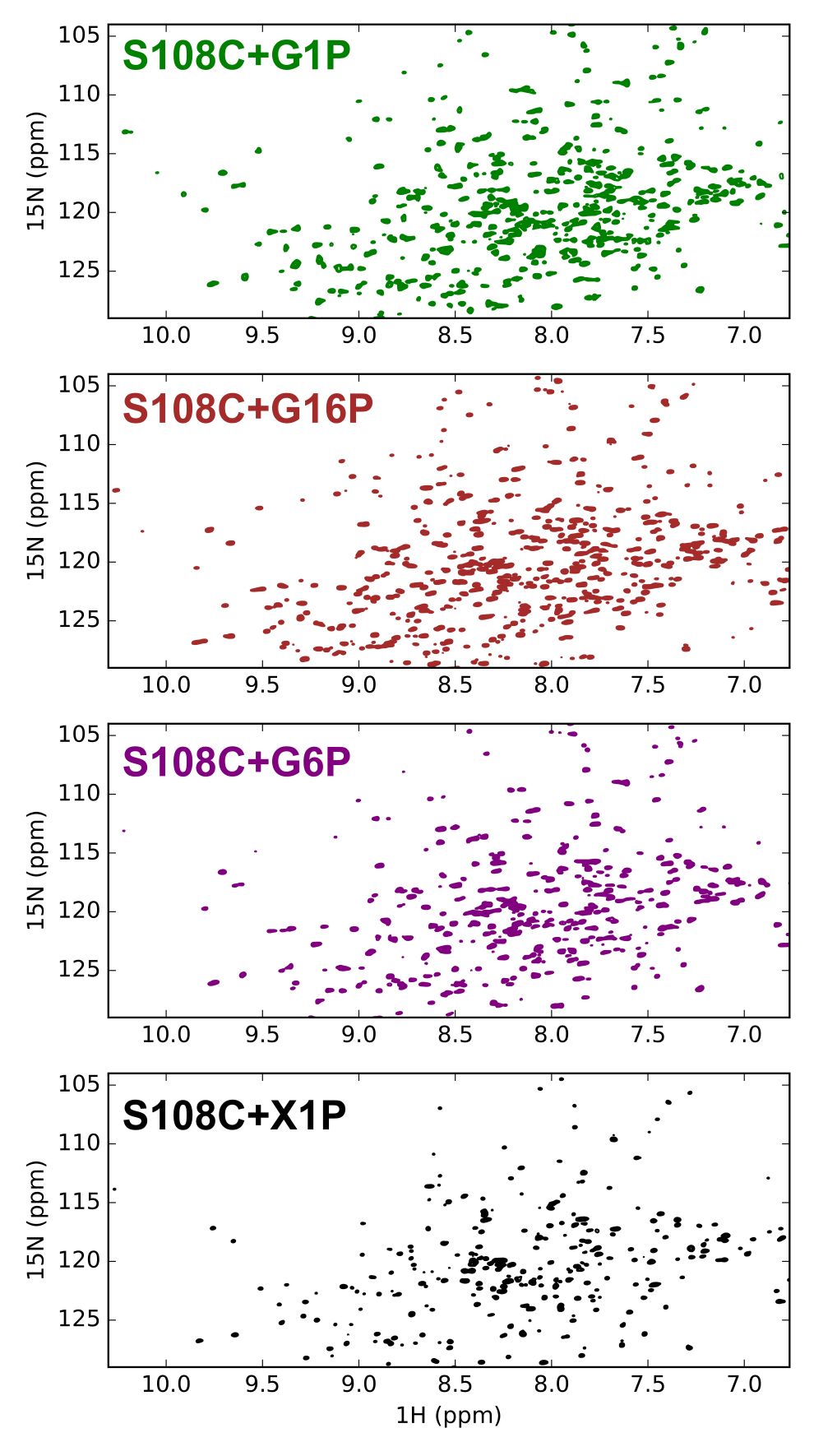
**

**Figure S3. ^15^N TROSY spectra of PMM/PGM(S108C) at 310 K with additions of G1P (green), G16P (brown), G6P (purple), or X1P (black).**


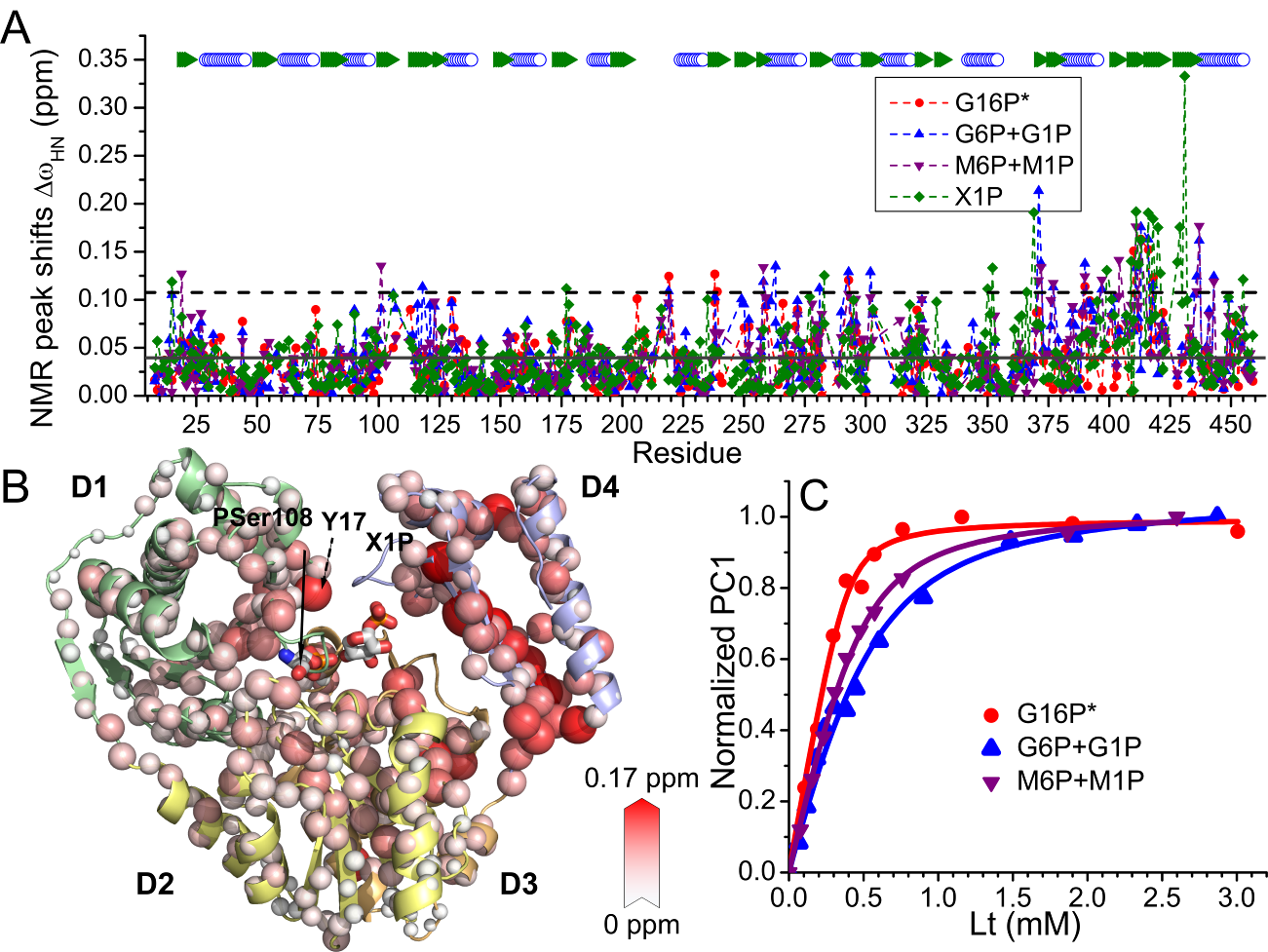


**Figure S4. Ligand binding perturbations of amide NMR peak positions of wt PMM/PGM.** (A) Plot of ^1^H/^15^N NMR peak shifts (the radial changes calculated per Eq. 1) introduced by additions of X1P, G16P, G6P, or M6P (suspected to be transformed in part to M1P). The enzyme activity upon G6P results in a steady state with G1P present at around one-third the concentration of G6P ^8^. Similarly, the M6P should be transformed in part to M1P. The * refers to the likelihood that the G16P was also transformed in part to G6P and G1P. (B) Locations of NMR peak shifts introduced by ligand binding are marked with spheres on the crystal structure of the complex with G16P (PDB ID: 2FKM). The magnitude of NMR peak shifts is marked by the white to red color gradient, as well as by the radii of the spheres. (C) The titrations of wt PMM/PGM, which yielded mixtures of phosphosugars, are plotted as a function of the total concentration of ligand added. Fitting of an apparent *K_D_* to the titration with G16P suggests that wt PMM/PGM binds G16P with affinity that is at least 10-fold greater than that of apparent *K_D_* for S108C-inactivated PMM/PGM reported in Figure 3.


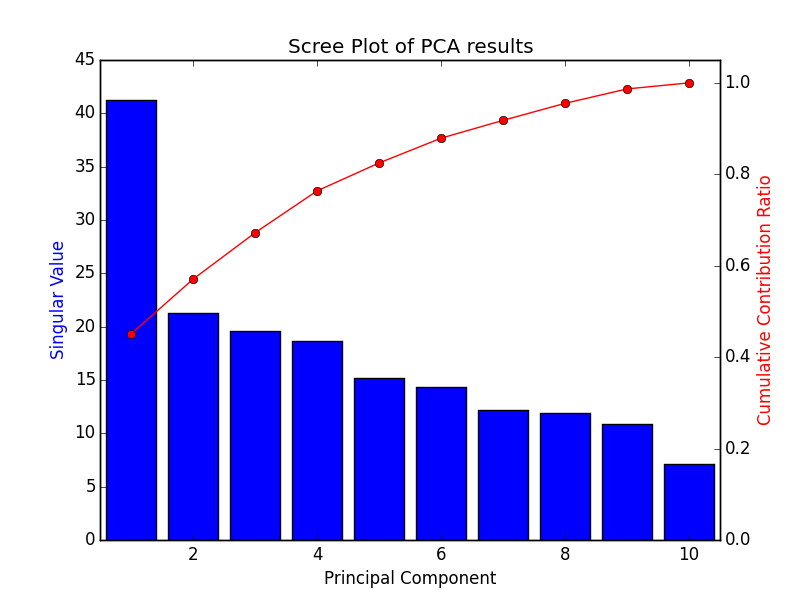


**Figure S5. Scree plot accompanying PCA biplots of Figure 5G, H.**


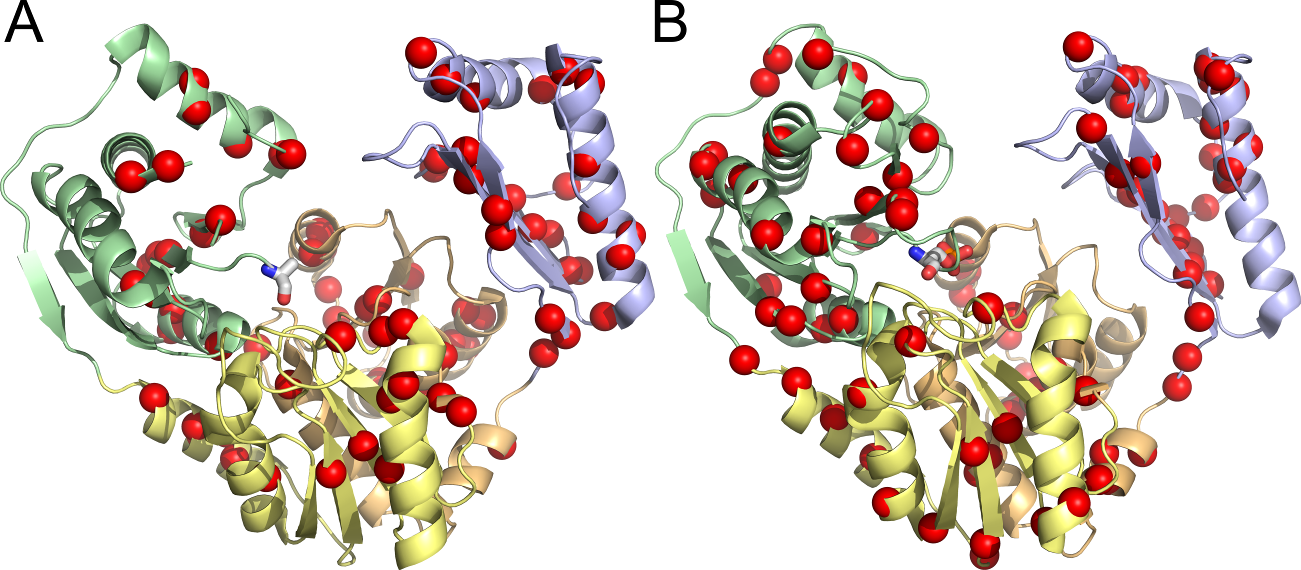


**Figure S6. The residues with amide NMR peaks shifted linearly by the ligands (red spheres) are distributed widely among the four domains of (A) PMM/PGM(S108C) and (B) wt PMM/PGM.** These amide peaks were used for the CONCISE analyses depicted in Figure 7. The crystal structures have PDB accession codes of 3RSM for PMM/PGM(S108C) and 1K35 for E_P_, i.e. wt PMM/PGM.


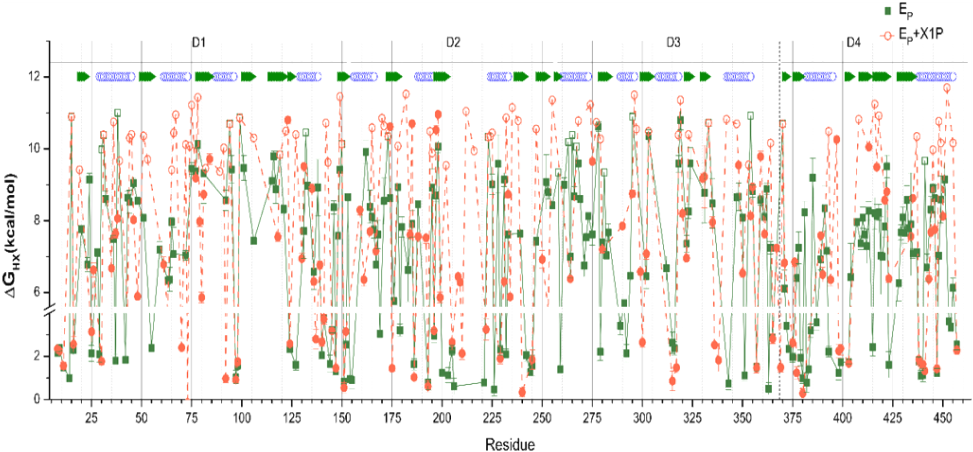


**Figure S7. Comparison of ΔG_HX_ between E_P_ and E_P_+X1P complex shows ligand-induced stabilization.** ΔG_HX_ values obtained by HDX experiments are shown above the break, while the small ΔG_HX_ values measured by CLEANEX-PM NMR are shown below the break. Lower bounds of the ΔG_HX_ that were estimated for slowly exchanging amide residues were shown in open squares. Strands and helices are marked by green triangles and blue circles at the top.

Supplementary References

1 Sarma, A. V. *et al.* Solution NMR of a 463-residue phosphohexomutase: domain 4 mobility, substates, and phosphoryl transfer defect. *Biochemistry* **51**, 807-819, doi:10.1021/bi201609n (2012).

2 Lee, Y., Villar, M. T., Artigues, A. & Beamer, L. J. Promotion of enzyme flexibility by dephosphorylation and coupling to the catalytic mechanism of a phosphohexomutase. *J. Biol. Chem.* **289**, 4674-4682, doi:10.1074/jbc.M113.532226 (2014).

3 Xu, J., Lee, Y., Beamer, L. J. & Van Doren, S. R. Phosphorylation in the catalytic cleft stabilizes and attracts domains of a phosphohexomutase. *Biophys. J.* **108**, 325-337, doi:10.1016/j.bpj.2014.12.003 (2015).

4 Xu, J. & Van Doren, S. R. Binding Isotherms and Time Courses Readily from Magnetic Resonance. *Anal. Chem.* **88**, 8172-8178, doi:10.1021/acs.analchem.6b01918 (2016).

5 Lee, Y., Furdui, C. & Beamer, L. J. Data on the phosphorylation state of the catalytic serine of enzymes in the alpha-D-phosphohexomutase superfamily. *Data Brief* **10**, 398-405, doi:10.1016/j.dib.2016.12.017 (2017).

6 Hwang, T. L., van Zijl, P. C. & Mori, S. Accurate quantitation of water-amide proton exchange rates using the phase-modulated CLEAN chemical EXchange (CLEANEX-PM) approach with a Fast-HSQC (FHSQC) detection scheme. *J. Biomol. NMR* **11**, 221-226, doi:10.1023/A:1008276004875 (1998).

7 Bertini, I., Ghosh, K., Rosato, A. & Vasos, P. R. A high-resolution NMR study of long-lived water molecules in both oxidation states of a minimal cytochrome c. *Biochemistry* **42**, 3457-3463, doi:10.1021/bi0272961 (2003).

8 Naught, L. E. & Tipton, P. A. Formation and reorientation of glucose 1,6-bisphosphate in the PMM/PGM reaction: transient-state kinetic studies. *Biochemistry* **44**, 6831-6836, doi:10.1021/bi0501380 (2005).
